# HDAC11 deficiency regulates age-related muscle decline and sarcopenia

**DOI:** 10.1007/s11357-025-01611-y

**Published:** 2025-04-12

**Authors:** Renato Odria, Aina Cardús, Clara Gomis-Coloma, Marta Balanyà-Segura, Alexandra Mercado-Amarilla, Pau Maestre-Mora, Andrea Poveda-Sabuco, Joan Carles Domingo, Gisela Nogales-Gadea, Jose A. Gomez-Sanchez, Erica Hurtado, Mònica Suelves

**Affiliations:** 1 Grup de Recerca en Malaties Neuromusculars de Badalona (GRENBA), Institut d’Investigació en Ciències de la Salut Germans Trias i Pujol (IGTP), 08916 Badalona, Spain; 2 Programa de Doctorat en Biomedicina, Universitat de Barcelona, 08007 Barcelona, Spain; 3 Universidad Católica de Valencia San Vicente Mártir, 46001 Valencia, Spain; 4 Universitat Rovira i Virgili, Unitat d’Histologia i Neurobiologia (UHNeurob), Facultat de Medicina i Ciències de la Salut, 43201 Reus, Spain; 5 Instituto de Investigación Sanitaria y Biomédica de Alicante (ISABIAL), 03010 Alicante, Spain; 6 Department de Bioquímica i Biomedicina Molecular, Facultat de Biologia, Universitat de Barcelona, 08028 Barcelona, Spain; 7 Instituto de Neurociencias de Alicante UMH-CSIC, 03550 San Juan de Alicante, Spain

**Keywords:** HDAC11, Sarcopenia, Muscle atrophy, Skeletal muscle regeneration, Fatty acid oxidation, Omega-6/omega-3 fatty acid ratio

## Abstract

Sarcopenia, defined as the progressive loss of skeletal muscle mass and function associated with ageing, has devastating effects in terms of reducing the quality of life of older people. Muscle ageing is characterised by muscle atrophy and decreased capacity for muscle repair, including a reduction in the muscle stem cell pool that impedes recovery after injury. Histone deacetylase 11 (HDAC11) is the newest member of the HDAC family and it is highly expressed in skeletal muscle. Our group recently showed that genetic deficiency in HDAC11 increases skeletal muscle regeneration, mitochondrial function and globally improves muscle performance in young mice. Here, we explore for the first time the functional consequences of HDAC11 deficiency in old mice, in homeostasis and during muscle regeneration. Aged mice lacking HDAC11 show attenuated muscle atrophy and postsynaptic fragmentation of the neuromuscular junction, but no significant differences in the number or diameter of myelinated axons of peripheral nerves. Maintenance of the muscle stem cell reservoir and advanced skeletal muscle regeneration after injury are also observed. HDAC11 depletion enhances mitochondrial fatty acid oxidation and attenuates age-associated alterations in skeletal muscle fatty acid composition, reducing drastically the omega-6/omega-3 fatty acid ratio and improving significantly the omega-3 index, providing an explanation for improved muscle strength and fatigue resistance and decreased mortality. Taken together, our results point to HDAC11 as a new target for the treatment of sarcopenia. Importantly, selective HDAC11 inhibitors have recently been developed that could offer a new therapeutic approach to slow the ageing process.

**Supplementary Information:**

The online version contains supplementary material available at 10.1007/s11357-025-01611-y.

## Introduction

Skeletal muscle is the largest tissue in mammals, and it contributes to multiple bodily functions such as voluntary movement, respiration, metabolism, and body homeostasis. During ageing there is a progressive loss of muscle mass and function, a process known as sarcopenia, which leads to a reduction in the quality of life of older people and an increase in their level of dependency [1]. Sarcopenia not only involves the loss and atrophy of muscle mass, but it is also characterised by the infiltration of fatty and fibrotic tissue and a reduced capacity for muscle repair; as the muscle stem cell pool is reduced, recovery after injury in older people is impaired. Skeletal muscles are connected to nerves, which receive information from the nervous system via the neuromuscular junctions (NMJs). With ageing, the morphology and density of NMJs change, and alterations in NMJs are increasingly linked to the onset and progression of sarcopenia [2]. In addition, during ageing, biochemical and morphological changes occur in the biology of Schwann cells, which are less favourable or more harmful to neurons and synapses, and in the myelination of peripheral nerves, affecting muscle function [3].

Skeletal muscle fatty acid composition varies with age, showing an increase in monounsaturated fatty acids (MUFA) with the corresponding decrease in saturated and polyunsaturated fatty acids (SFA and PUFA, respectively) [4]. The two main classes of PUFAs are the essential omega-3 and omega-6 fatty acids, and a balanced ratio of omega-6 to omega-3 is a critical factor for lifelong health, with a high ratio of omega-6 to omega-3 found to be linked to the pathogenesis of many diseases and ageing [5, 6].

The world’s population is currently shifting towards an older median age. It is estimated that by the year 2050 almost a quarter of the world’s population will be over 65 years of age, which will have a significant financial and social impact worldwide[7]. At present, age-related sarcopenia treatments are limited to exercise therapy, nutritional intervention, and some therapeutic drugs with limited efficacy [8, 9]. Therefore, it is essential to better understand the mechanisms of age-related muscle loss to develop therapeutic interventions to slow muscle functional decline and extend healthy ageing.

Histone deacetylases (HDACs) are proteins that catalyse the removal of acetyl groups (deacetylation) from e-lysine residues of numerous substrate proteins, including histones, transcription factors and signalling proteins. Therefore, HDAC family proteins regulate a variety of cellular processes at multiple levels including gene expression, transcription factor activity, cell signalling pathways, and protein degradation [10]. In mammals there are 18 distinct HDACs categorized into two families: the classical HDACs (class I, II and IV), which employ zinc as a co-factor for catalytic activity, and the sirtuins (class III), which require nicotinamide adenine dinucleotide (NAD^+^) for enzymatic function [10]. Interestingly, it has been shown that HDAC inhibitors (HDACi) have the potential to counteract multiple aspects of ageing, although the role of each HDAC has not been deeply addressed thus far [11].

HDAC11 was the latest HDAC discovered. Since it was sufficiently phylogenetically different from the other HDACs, a separate class, called class IV, was created and it remains its sole member. Intriguingly, recent studies showed that HDAC11 is not a conventional HDAC, but is a multifaceted enzyme with highly efficient long fatty acid deacylation activity, displaying the most efficient and specific demyristoylation activity described to date [12–14]. HDAC11 is highly expressed in skeletal muscles and our group recently showed that genetic deficiency in HDAC11 in young mice increases skeletal muscle regeneration, mitochondrial function and globally improves muscle performance [15, 16]. Here, we have explored for the first time the functional consequences of HDAC11 deficiency in ageing, specifically focused in skeletal muscle homeostasis and during muscle regeneration. Remarkably, our results show that old mice lacking HDAC11 show diminished mortality, attenuated muscle atrophy and ageing-associated postsynaptic fragmentation of the NMJ, increased muscle regeneration, and maintenance of the muscle´s stem cell reserve. HDAC11 depletion enhances muscular mitochondrial fatty acid oxidation and attenuates age-associated alterations in skeletal muscle fatty acid composition. Importantly, at the functional level, old mice deficient in HDAC11 show increased fatigue resistance and muscle strength, resulting in globally improved muscle function. Altogether, our results highlight HDAC11 as a novel target for the treatment of sarcopenia. Importantly, selective HDAC11 inhibitors have recently been developed that could offer a new therapeutic approach to slow the ageing process.

## Results

### HDAC11 deficiency reduces mortality, prevents ageing-associated muscle wasting, and attenuates atrophy of type II fibers in aged muscles

**Fig. 1:**
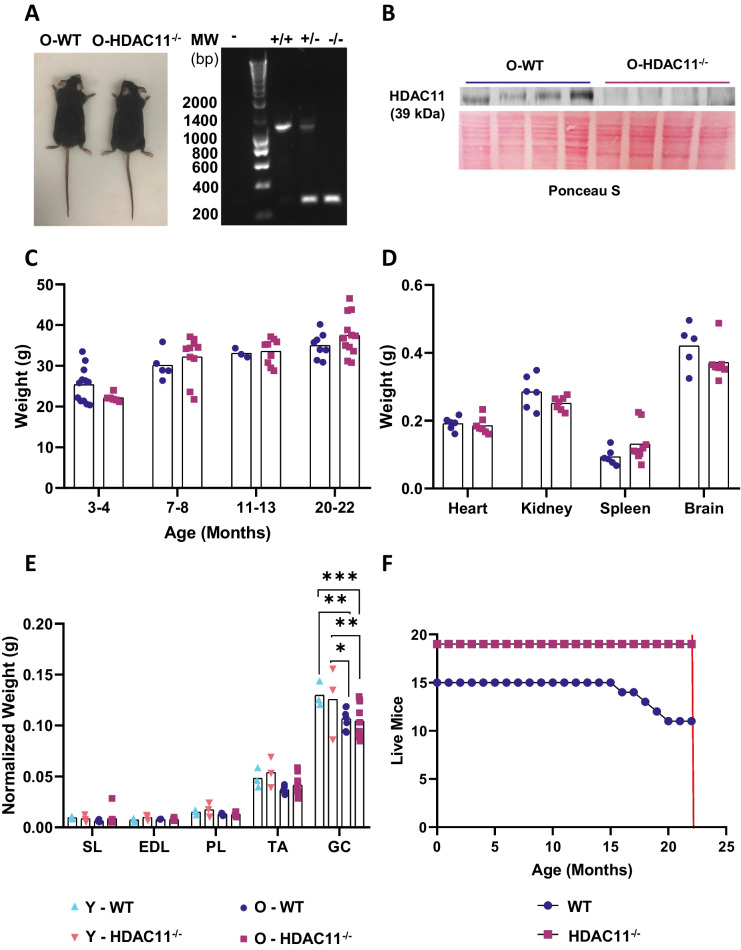
HDAC11 deficiency does not alter the physical characteristics of old mice, but reduces their mortality. **A** Representative image of an old WT mouse and an old HDAC11^−/−^ mouse (left) and confirmation of mice genotypes by PCR (right). **B** WB analysis of HDAC11 in tissue from old WT (O-WT) and old HDAC11^−/−^ mice (O-HDAC11^−/−^). **C** Weight of male and female WT and HDAC11^−/−^ mice at different ages.** D** Weight of tissues were HDAC11 is highly expressed in WT and HDAC11^−/−^ old male mice. **E** Weight of muscles normalized to body weight of WT and HDAC11^−/−^ young and old male and female mice. **F** Survival of WT and HDAC11^−/−^ male and female mice until date of euthanasia (represented by red line). Data information: each dot represents a different mouse. n = 15 for WT mice and n = 19 for HDAC11^−/−^ mice in panel F. Young mice are between 3–4 months of age and old mice are between 20–22 months of age. In (B), + / + denotes a WT mouse, +/- a mouse heterozygous for HDAC11 and -/- an HDAC11 KO mouse. SL (Soleus), EDL (extensor digitalis longus), PL (plantaris), TA (tibialis anterior), and GC (gastrocnemius). Statistical significance was determined by two-tailed Student’s t-test and two-way ANOVA

To investigate the consequences of HDAC11 deficiency in ageing, total *Hdac11* knockout mice (HDAC11^−/−^) were left to age for 2 years. HDAC11 loss was assessed by genotyping and western blot of protein extracts (Fig. 1A-B). Old WT and HDAC11^−/−^ mice were phenotypically indistinguishable; showed no differences in body, organs, and skeletal muscle measurements (Fig. 1C-E), and showed no compensation of other HDAC members (Figure S1). However, HDAC11^−/−^ mice showed a lower mortality rate. Twenty-seven percent of the WT mice (irrespective of sex) died before 22 months due to spontaneous death, age-associated tumours, as well as other pathologies that resulted in premature euthanasia recommended by the veterinary staff, while none of the HDAC11^−/−^ mice died before their date of euthanasia that was established at 22 months (Fig. 1F).

**Fig. 2:**
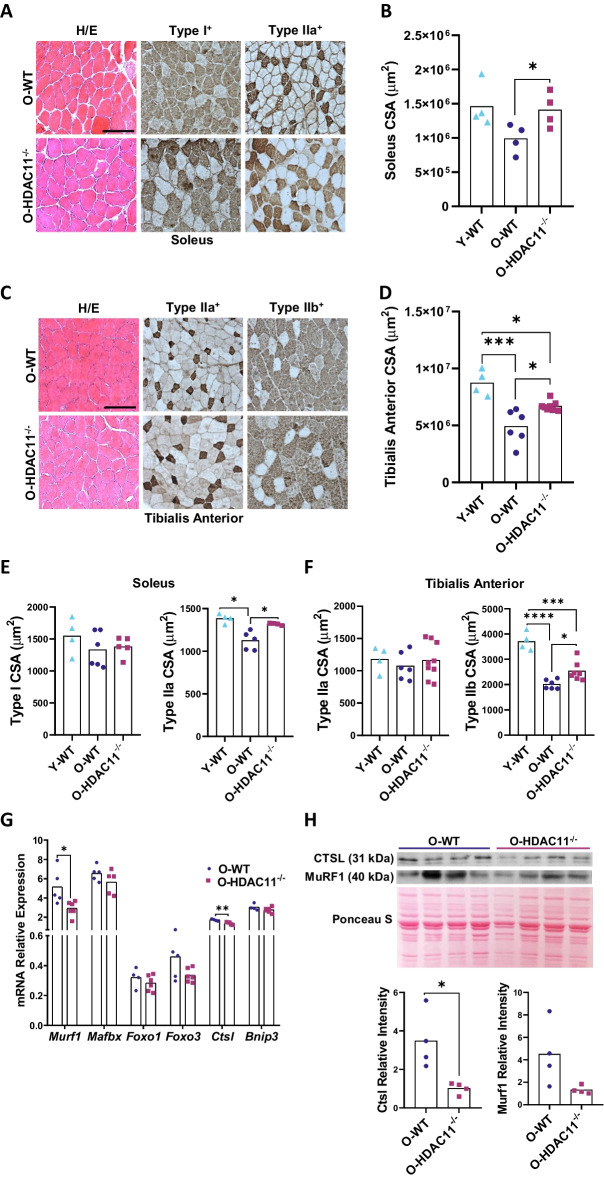
HDAC11 deficiency prevents loss of muscle mass and attenuates fiber-specific atrophy in aged soleus and tibialis anterior muscle. **A** Left: hematoxylin/eosin (H/E), middle: MyHC-I immunostaining (type I) and right: MyHC-IIa immunostaining (type IIa) representative sections of soleus muscle from old wildtype (O-WT) and old HDAC11^−/−^ (O-HDAC11^-/-^) mice. **B** Quantification of soleus midbelly cross-sectional area (CSA) of young WT mice (Y-WT), old WT mice (O-WT), and old HDAC11^−/−^ mice (O-HDAC11^−/−^). **C** Left: hematoxylin/eosin (H/E), middle: MyHC-IIa immunostaining (type IIa) and right: MyHC-IIb immunostaining (type IIb) representative sections of tibialis anterior (TA) muscle from old WT and HDAC11^−/−^ mice. **D** Quantification of TA midbelly CSA of young WT mice (Y-WT), old WT mice (O-WT), and old HDAC11^−/−^ mice (O-HDAC11^−/−^). **E** Quantification of type I fiber CSA (left) and type IIa fiber CSA (right) in aged soleus muscle of young WT mice (Y-WT), old WT mice (O-WT), and old HDAC11^−/−^ mice (O-HDAC11^−/−^). **F** Quantification of type IIa fiber CSA (left) and type IIb fiber CSA (right) in aged TA muscle from young WT mice (Y-WT), old WT mice (O-WT), and old HDAC11^−/−^ mice (O-HDAC11^−/−^). **G** Quantification of mRNA levels of ubiquitin proteasomal (MurF1 and MAFbx), autophagic-lysosomal (CTSL and Bnip3) related atrogenes and FOXO transcription factors by RT-qPCR of TA muscle from young WT mice (Y-WT) and old WT mice (O-WT). **H** WB analysis of CTSL1 and MuRF1 normalized with Ponceau S staining (top) and quantitative analysis (bottom) in muscle from old WT and old HDAC11^−/−^ mice**.** The scale bar represents 100 µm. Data information: young mice are between 3–4 months of age and old mice are between 20–22 months of age. Each dot in the graphs represents a different mouse. For the CSA quantification a minimum of 200 positive fibers were analyzed per animal. Statistical significance was determined using ANOVA in A-G and two-tailed Student’s t-test in H (*p < 0.05; **p < 0.01, ***p < 0.001; ****p < 0.0001)

To evaluate the consequences of HDAC11 deficiency on skeletal muscle architecture, we conducted a detailed analysis of soleus (SL) and tibialis anterior (TA) muscles, as examples of slow and fast muscles, respectively. The haematoxylin and eosin (H/E)-stained cryosections of aged muscles did not show any morphological difference at structural level between genotypes (Figs. 2A and C). However, when we measured the cross-sectional area (CSA) of the entire muscles at the mid belly, aged HDAC11^−/−^ mice showed an attenuated age-associated reduction in CSA of both soleus and TA muscles (Figs. 2B and D). Remarkably, when we compared these measurements with the CSA of SL and TA muscles from young WT animals, we observed that the area of aged HDAC11^−/−^ muscles more closely resembles the CSA of young muscles, showing a preservation of the muscle mass. To investigate whether HDAC11 deficiency had an impact on the size of the different fiber types, we quantified the cross-sectional area of type I and type IIa fibers in soleus muscles and type IIa and type IIb fibers in TA muscles, after staining the different fiber types with specific antibodies (Figs. 2A and C).

As shown in Fig. 2E-F, HDAC11 deficiency significantly attenuates muscle atrophy of type IIa fibers in the soleus and type IIb fibers in the tibialis anterior, which are the fibers showing significant size reduction between young and old WT mice. Type IIa fibers of old HDAC11^−/−^ soleus muscles are almost 20% larger than old WT fibers, while type IIb fibers of old HDAC11^−/−^ tibialis anterior muscles are 26% larger than old WT fibers. Myofiber size distribution confirmed the higher proportion of larger myofibers in aged HDAC11-deficient muscles compared to WT ones (Figure S2). Overall, our results suggest that absence of HDAC11 reduces ageing-associated muscle wasting and attenuates type II myofiber atrophy in aged muscles.

Muscle atrophy involves an imbalance in protein synthesis and degradation and is mediated by several genes collectively referred to as atrogenes, which include members of the ubiquitin–proteasome and the autophagy-lysosomal systems [17]. We analyzed the expression by qRT-PCR of a set of atrogenes (Fig. 2G) including the autophagic/lysosomal related gene Ctsl (Cathepsin L1) and the E3 ubiquitin-protein ligase MuRF1 (Muscle Ring Finger 1). As shown in Fig. 2G both genes are less expressed in old HDAC11^−/−^ muscles, and lower levels of CTSL1 and MuRF1 protein were found (Fig. 2H), suggesting that HDAC11 deficiency would have an impact on muscle loss related mechanisms.

### HDAC11 deficiency reduces NMJ postsynaptic fragmentation in aged muscles

**Fig. 3:**
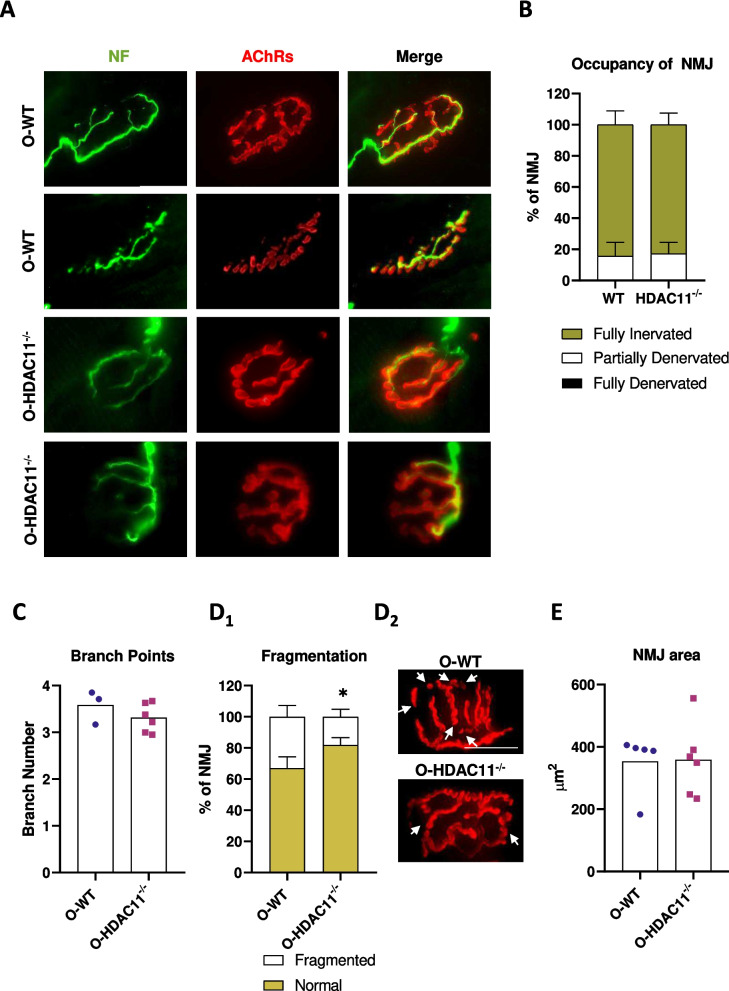
NMJ morphological analysis of muscles from old WT and old HDAC11^−/−^ mice. **A** Representative images of NMJs from old WT (O-WT) and old HDAC11^−/−^ (O- HDAC11^−/−^) quadriceps doubly labeled with neurofilament (NF, green) and α-bungarotoxin (AChRs, red) to identify the presynaptic nerve terminal and the postsynaptic end plate, respectively. Quantification of the percentage of postsynaptic component occupancy by the NF **B**, number of branching points **C**, NMJ fragmentation quantification **D**_**1**_ and representative image per genotype **D**_**2**_, and the postsynaptic NMJ area **E**. The scale bar represents 25 µm. Data information: old mice are between 20–22 months of age and each dot in graphs c and e represents a different mouse. In b and d, data are represented as means ± SD. In the images in d, the white arrows mark the discontinuous fragments of AChR-rich membranes. n = 3–6 mice per genotype. Statistical significance was determined by two-tailed unpaired Student's t-test (*p < 0.05)

To examine whether HDAC11 could be involved in the structural organization of aged neuromuscular junctions (NMJs) we studied the NMJ morphology and analysed structural characteristics like the number of branching points, the postsynaptic NMJ area and its fragmentation to determine the percentage of NMJ denervation. The NMJ pre- and postsynaptic components from old muscles were labelled with neurofilament (as a presynaptic marker to label motor nerve axons) and α-bungarotoxin (a selective and potent ligand for AChR, to visualize the postsynaptic membrane). As shown in Fig. 3A, there was no obvious qualitative difference between the morphological appearance of the NMJs from WT and HDAC11^−/−^ old muscles. Next, we performed quantitative analysis measuring the percentage of NMJ denervated either partially or totally and no differences between genotypes were detected (Fig. 3B). The number of branching points were also analysed to detect changes which could be indicators of NMJ remodelling, and in muscles lacking HDAC11 we observed a slight reduction in branch points, without reaching statistical significance (Fig. 3C). However, analysis of the number of AChR-enriched membrane fragments in individual NMJs revealed a significant attenuation in the mean number of fragments in HDAC11^−/−^ mice compared to WT mice. The percentage of fragmented NMJs decreased from 30 ± 10% to 18 ± 6% in HDAC11^−/−^ aged mice (Fig. 3D), although, despite the decrease in the number of fragments, there was no significant change in the total area occupied by these fragments (Fig. 3E). These results would indicate that the lack of HDAC11 attenuates the postsynaptic fragmentation of the NMJ associated with ageing without affecting the organisation of the presynaptic parameters studied or the postsynaptic area of the NMJ.

### Normal peripheral nerves in aged HDAC11 deficient mice

**Fig. 4:**
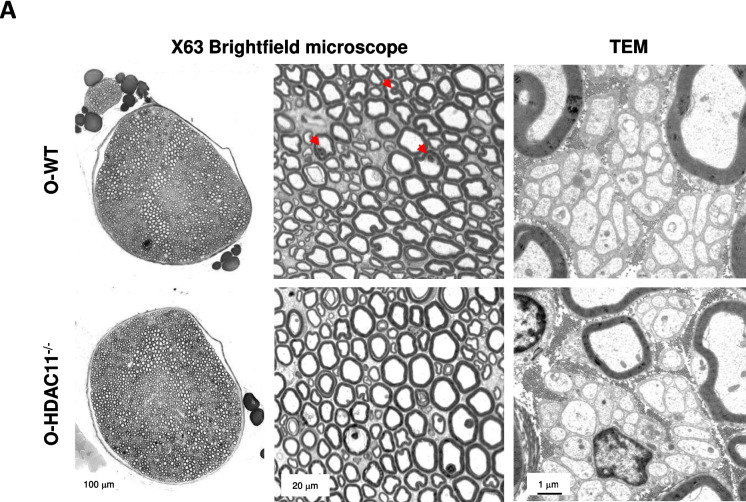

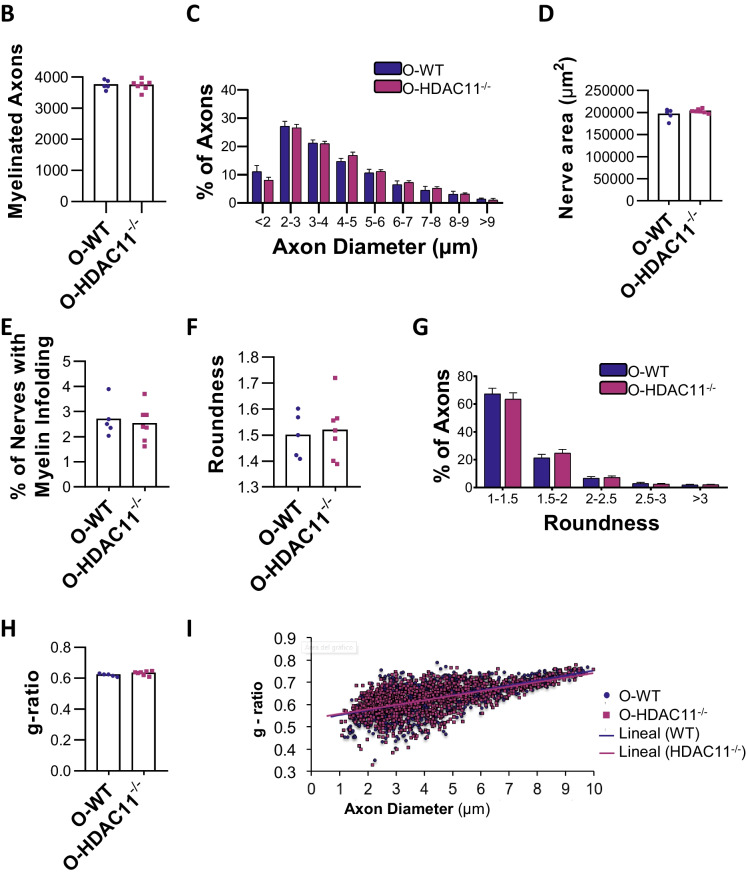
Aged HDAC11^−/−^ sciatic nerves present normal ultrastructure and myelination without apparent abnormalities. **A** Representative mosaic of the sciatic nerve and myelinated axons obtained from semi-thin cross-sections at × 63 magnification using a brightfield microscope, along with Remak bundles containing unmyelinated nerves captured from ultrathin sections using transmission electron microscopy (TEM). The images illustrate the sciatic nerve of old HDAC11^−/−^ and WT mice. Red arrows indicated myelin infoldings. Quantification of the myelinated axons **(B)**, axon diameter distribution **(C)**, sciatic nerve area **(D)**, myelin infoldings **(E)**, axon roundness **(F)**, axon diameter **(G)** *g-ratio* **(H)** of both WT (O-WT) and HDAC11^−/−^ (O- HDAC11^−/−^) old sciatic nerves. **I** Scatter plots depicting g-ratios of old WT (blue circles) and old HDAC11^−/−^ mice (purple squares) are fitted with a linear function. The scale bars represent 100 µm, 20 µm, and 1 µm. Data information: aged mice are between 20–22 months of age and each dot in the graphs represents a different mouse. n = 5–7 mice per genotype. Statistical significance was determined by two-tailed Student’s t-test in b, d, e, f and h, and ANOVA followed by Bonferroni correction in c and g

HDACs have recently been identified as critical regulators of myelin homeostasis and Schwann cell biology during ageing [18]. To investigate the potential role of HDAC11 in myelin maintenance and Schwann cell biology within aged nerves, we performed histological analyses of sciatic nerves to determine whether aged HDAC11 knock-out mice exhibit peripheral nerve abnormalities that could affect muscle phenotype. Initially, we performed a morphometric analysis of peripheral nerve myelination using semithin cross-sections of aged control and mutant mice to quantify the number of myelinated fibers in the sciatic nerve (Fig. 4A). Our analysis revealed no significant difference in various axon parameters between HDAC11^−/−^ and WT mice (Fig. 4B-G). Myelin infoldings are abnormal inward foldings of the myelin sheath into the axon, which can lead to pathological conditions or ageing [19]. The analysis of myelin infoldings (marked with a red arrow in Fig. 4A) showed that aged HDAC11 KO animals exhibited a similar amount compared to WT mice (Fig. 4E). Next, we assessed myelin thickness by calculating the g-ratio (the ratio of the axon diameter to the myelinated fiber diameter) on toluidine blue-stained semithin sections of aged sciatic nerves, but no evidence of hypo or hypermyelination in aged HDAC11^−/−^ mice compared to their controls (Fig. 4H-I). To further characterize any pathological issues in unmyelinated axons, we performed transmission electron microscopic (TEM) analyses of sciatic nerves from the mutant mice and control mice. The results revealed no abnormalities in Remak bundles of unmyelinated axons. The unmyelinated axons were efficiently segregated by the non-myelin Schwann cells within their distinct pockets, displaying a consistent pattern across all analysed samples (Fig. 4A).

Altogether, these ultrastructural data demonstrate the absence of obvious abnormalities in the number of myelinated axons, g-ratio, myelin infoldings, and axonal roundness of peripheral nerves in aged HDAC11^−/−^ mice.

### Aged HDAC11 knockout mice display better muscle performance than aged WT mice

**Fig. 5:**
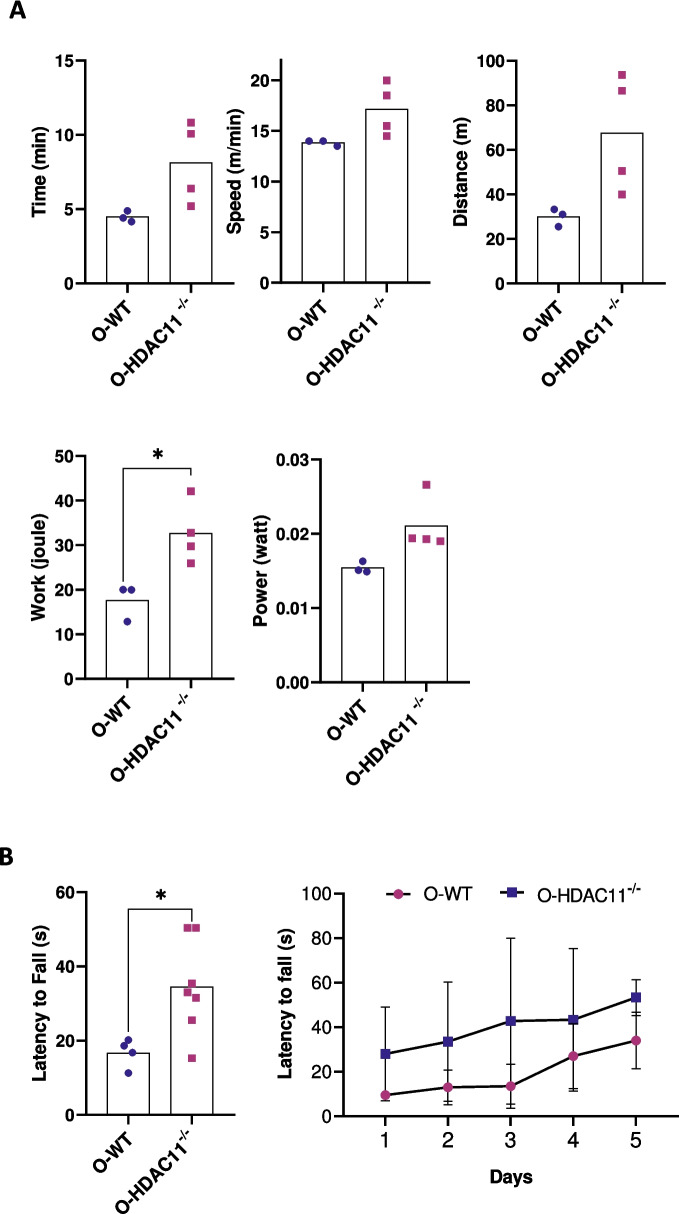
HDAC11 deficiency improves muscle endurance and strength. **A** Treadmill exhaustion test was performed with progressive speed with a 20% slope. Time, speed, and distance were quantified until exhaustion in old WT (O-WT) and old HDAC11^−/−^ (O-HDAC11^−/−^) mice. Work and power were normalized to individual body weight. **B** Kondziellas inverted screen test was performed in WT (O-WT) and HDAC11^−/−^ (O-HDAC11^−/−^) mice during 5 nonconsecutive days. Left: Average latency to fall. Right: maximum hanging time per day. Data information: each dot represents a different mouse. n = 3–7 mice per genotype. All mice are between 20–22 months of age. Statistical significance was determined by two-tailed Student’s t-test (*p < 0.05)

Our group recently reported that young HDAC11^−/−^ mice exhibited enhanced muscle performance than WT mice [16]. To test whether HDAC11 deficiency also improved muscle function during ageing, WT and HDAC11^−/−^ aged mice were submitted to increased exercise load on a treadmill machine, equipped with electronic shock grids. The treadmill had a progressive speed set at a 20% slope, and we measured the time until exhaustion. As shown in Fig. 5A, although the results did not reach significant differences, WT mice spent an average of 4.48 ± 0.36 min running on the treadmill, while HDAC11^−/−^ spent an average of 8.12 ± 2.75 min, a difference of more than 50%. Regarding distance, WT mice were able to achieve a distance ran of 29.92 ± 3.99 m while HDAC11^−/−^ mice ran 67.67 ± 26.4 m, an improvement of 75%. As well, the average top speed that WT mice reached was 13.8 ± 0.29 while HDAC11^−/−^ mice were able to reach an average top speed of 17.13 ± 2.56 (an improvement of 20%). Lastly, work and power parameters were calculated on weight and slope. WT mice were able to perform 17.6 ± 4.14 J of work, while HDAC11^−/−^ mice were able to perform 32.6 ± 6.90 J of work (a significant improvement of 77%). As well, WT mice generated 0.0154 ± 0.000757 W of power, while HDAC11^−/−^ generated 0.0211 ± 0.00369 W (an improvement of 32%).

We also performed the four limbs hang test (also known as Kondziella’s inverted grid test), which uses a wire grid to test how long a mouse can oppose gravity when suspended from the grid. HDAC11^−/−^ aged mice were able to spend an average of 34.5 ± 12.7 s while WT mice were only able to withstand an average of 16.7 ± 3.9 s, which is a significant increase of 106.5% (Fig. 5B). As well, over the span of the test days, HDAC11^−/−^ old mice consistently performed better, and stayed suspended from the grid for a longer time (Fig. 5B).

Taken together, these results demonstrate that aged mice lacking HDAC11 show enhanced endurance and strength, and improved muscle performance.

### Fatty acid metabolism is enhanced in aged HDAC11^−/−^ mice

**Fig. 6:**
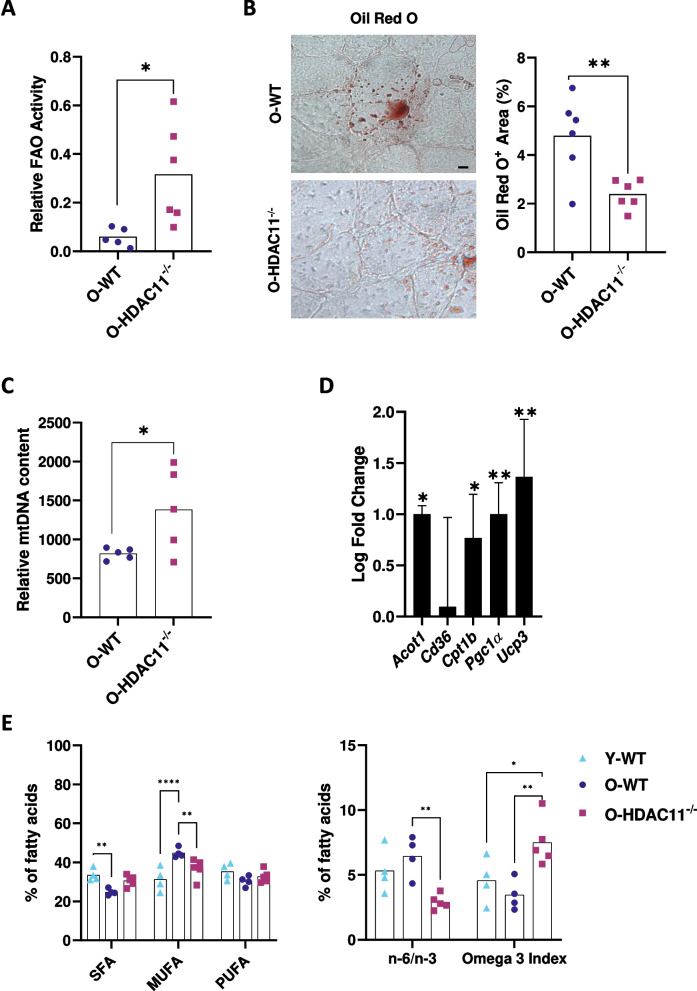
Fatty acid metabolism is enhanced in HDAC11^−/−^ old mice. **A** Measurement of fatty acid oxidation (FAO) activity in old WT (O-WT) and old HDAC11^−/−^ (O-HDAC11^−/−^) mice by measurement of INT-formazan by optical density (O.D. 492 nm). **B** Oil red representative image of the soleus muscle (left) and its quantification (right). **C** Quantification of mitochondrial DNA fragment COX2 by qPCR normalized to SDHA genomic DNA. **D** Quantification of FAO related genes by RT-qPCR expressed as log fold change relative to WT mice. **E** Fatty acids profile of young WT mice (Y-WT), old WT mice (O-WT), and old HDAC11^−/−^ mice (O-HDAC11^−/−^) depicting saturated fatty acids (SFA), mono unsaturated fatty acids (MUFA), and polyunsaturated fatty acids (PUFA) (left) and the ratio of omega-6 to omega-3 fatty acids (n-6/n-3) and the omega 3 index (right). The scale bar represents 10 µm. Data information: each dot represents a different mouse. n = 4–6 mice per genotype. The bars in D represent the standard deviation. Young mice are 3–4 months old and old mice are between 20–22 months of age. Statistical significance was determined by two-tailed Student’s t-test in a-d and ANOVA in E. (*p < 0.05, **p < 0.01)

Our group recently demonstrated increased capacity of fatty acid oxidation (FAO) in young HDAC11^−/−^ muscles [16], suggesting that HDAC11 could be an important regulator of lipid metabolism. In aged muscle, FAO activity showed a significant increase in mice lacking HDAC11 compared to WT (Fig. 6A). In addition, muscle cross-sections stained with Oil Red O showed significantly fewer lipid droplets (2.4% ± 0.6) in older HDAC11^−/−^ soleus compared to WT muscles (5.3% ± 1.1) (Fig. 6B).

Next, we analysed the mitochondrial content in these muscles by measuring mitochondrial DNA levels (mt-DNA). As shown in Fig. 6C, HDAC11^−/−^ old muscles showed an increase in mitochondrial DNA (*Cox2*) relative to genomic DNA (*Sdha*), which could correlate with a higher mitochondrial content, that may enhance their oxidative capacity. We analysed the expression of a panel of genes related to FAO by RT-qPCR, including *Acot1* (Acyl-CoA thioesterase 1, which catalyses the hydrolysis of Acyl-CoAs), *Cd36* (cluster of differentiation 36 that is a scavenger receptor for long fatty acid uptake), *Cpt1b* (carnitine palmitoyl transferase 1 that promotes the entry of fatty acids into the mitochondria), *Pgc1α* (peroxisome proliferator-activated receptor gamma coactivator 1 alpha, the master regulator of mitochondrial biogenesis that also upregulates expression of genes in the mitochondrial FAO pathway), and *Ucp3* (uncoupling protein 3 involved in many functions related to fatty acid shuffling). As shown in Fig. 6D, all genes except *Cd36* were significantly more than 70% expressed in HDAC11^−/−^ old muscles, suggesting that HDAC11 may be an important regulator of lipid metabolism by increasing the fatty acid oxidation capacity of muscle cells when deficient.

We determined the composition of fatty acids in young and old muscles by GC–MS (Gas Chromatography-Mass Spectrometry) analyses and as shown in Fig. 6E, muscle fatty acid composition varies with age, showing an increase in monounsaturated fatty acids (MUFA) with the corresponding decrease in saturated and polyunsaturated fatty acids (SFA and PUFA, respectively). These changes were associated with an increase in the omega-6/omega-3 ratio and a decrease in the omega 3 index. Interestingly, in old HDAC11^−/−^ muscles these changes were attenuated (Fig. 6E), showing similar fatty acid composition to young WT muscles. In addition, and importantly, the significant reduction in the omega-6/omega-3 ratio and the dramatic increase in the omega 3 index, may improve muscle function and skeletal muscle metabolism in HDAC11^−/−^ mice. Similar changes in fatty acid composition were observed in young HDAC11^−/−^ muscles (Figure S3).

### HDAC11 deficiency protects satellite cell pool depletion and improves skeletal muscle regeneration upon injury

**Fig. 7:**
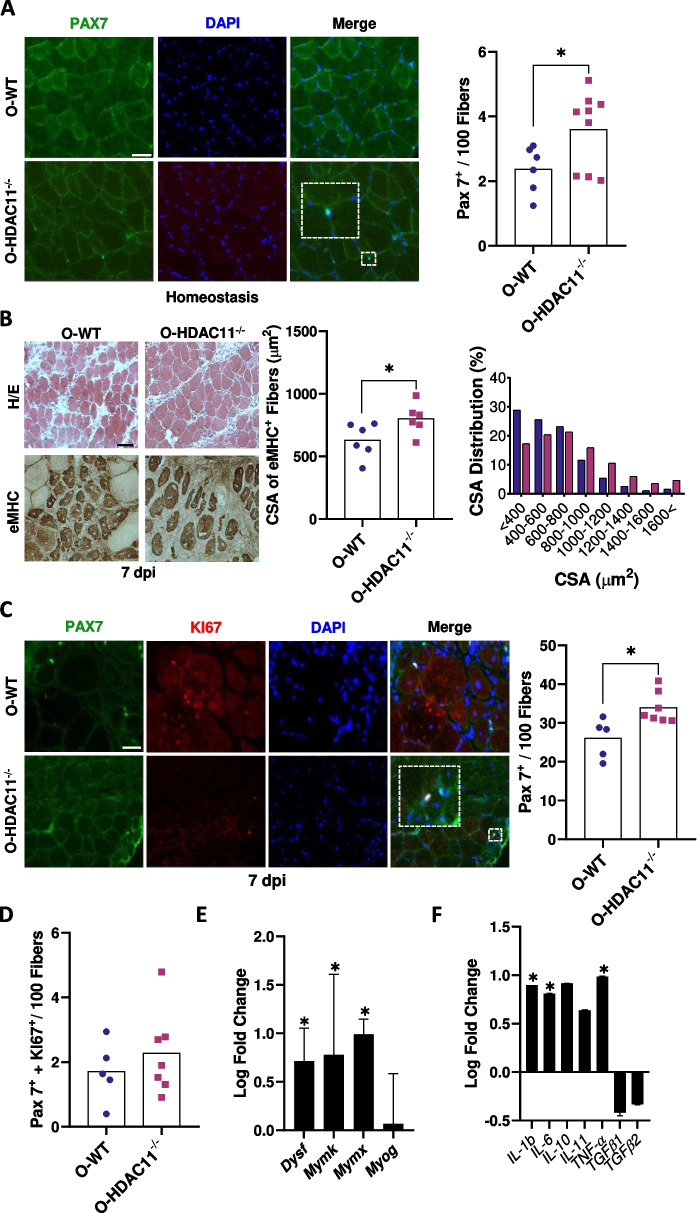
Skeletal muscle regeneration is accelerated in aged HDAC11^−/−^deficient mice. **A** Left: representative sections of tibialis anterior muscles from old WT (O-WT) and old HDAC11^−/−^ (O-HDAC11^−/−^) mice stained with PAX7 (green) and DAPI (blue). Right: quantification of PAX7^+^ SC number per 100 fibers in each muscle. **B** Left: Hematoxylin/eosin (H/E) representative sections (top) and embryonic myosin heavy chain (eMYHC) staining (bottom) to visualize regenerating fibers at 7 days post injury (dpi) of tibialis anterior (TA) muscles from WT (O-WT) and HDAC11^−/−^ (O-HDAC11^−/−^) old male mice. Right: average cross-sectional area (CSA) of eMYHC-positive myofibers at 7dpi and their distribution. **C** Left: Representative sections of TA muscles from WT (O-WT) and HDAC11^−/−^ (O-HDAC11^−/−^) old mice stained with PAX7 (green), KI67 (red), and DAPI (blue). Right: Quantification of PAX7^+^ SCs per 100 central nucleated fibers (CNFs). **D** Quantification of PAX7^+^/ Ki67^+^ SCs per 100 CNFs of WT (O-WT) and HDAC11^−/−^ (O-HDAC11^−/−^) TA aged muscle. Relative expression of repair- and fusion-related genes **E** and inflammatory genes **F** expressed as log fold change of HDAC11.^−/−^ samples versus WT samples. The scale bar represents 50 μm. Data information: Each dot in the graphs represents a different mouse. n = 5–7 mice per genotype. All mice are between 20–22 months of age. In a, a minimum of 400 myofibers were analyzed. In b-d, a minimum of 200 CNFs were analyzed per muscle and animal using at least five random images per animal. In **E** and **F** a minimum of 5 different mice per group were analyzed. The bars in E and F represent the standard deviation. The dotted boxes represent a magnified section of the image. Statistical significance was determined by two-tailed Student’s t-test; (*p < 0.05)

One of the hallmarks of ageing is stem cell exhaustion, which implies reduced tissue renewal rate and reduced regeneration capacity. In skeletal muscle, the satellite cells (SCs) are the main drivers of muscle regeneration. Satellite cells decline in number and in function with ageing, which results in lower muscle mass and in reduced capability for muscles to regenerate upon injury [20, 21]. To assess whether HDAC11 deficiency affected the satellite cell pool in homeostasis during ageing, we tagged satellite cells with the paired box transcription factor PAX7 (marker of SCs) and PAX7^+^/DAPI^+^ cells were quantified in TA and gastrocnemius (GC) muscles of both genotypes. Notably, we observed a significant increase in the number of SCs in old TA muscles deficient for HDAC11, where WT mice had a mean of 2.4 ± 0.7 SCs per 100 fibers and HDAC11^−/−^ old mice had a mean of 3.6 ± 1.2 SCs per 100 fibers (Fig. 7A). These results were confirmed in GC muscles, where the number of SCs was also significantly higher in mice lacking HDAC11(WT GC had an average of 2.5 ± 0.5 SCs per 100 fibers while HDAC11^−/−^ GC had an average of 3.8 ± 0.5 SCs per 100 fibers) (Figure S4). Importantly, HDAC11^−/−^ SCs were negative for MYOD and KI67 immunostaining (data not shown), confirming their quiescent status in homeostasis. These results show that the absence of HDAC11 protects the muscle stem cell pool and attenuates satellite cell depletion during ageing.

We then comparatively analysed the regenerative capacity of skeletal muscle from old HDAC11^−/−^ mice in response to intramuscular injection of cardiotoxin (CTX), a well-established model of acute muscle injury, at 7 days post injury (dpi), which represents an intermediate point in the kinetics of muscle regeneration. Sections of injured TA muscles from old WT and HDAC11^−/−^ mice were stained with haematoxylin and eosin (H/E) and embryonic myosin heavy chain (eMHC) to identify and measure the CSA of regenerating myofibers. As shown in Fig. 7B, the mean size of eMYHC^+^ myofibers was significantly larger in old HDAC11^−/−^ mice compared to WT mice, and the size distribution of regenerating myofibers (eMHC^+^ fibers) showed smaller number of smaller fibers and larger number of larger fibers in HDAC11-deficient muscles (Fig. 7B).

Next, we examined the basis of the advanced muscle regeneration process in the absence of HDAC11 by counting the total number of PAX7^+^ cells and the number of proliferating SCs (PAX7^+^/KI67^+^) at 7dpi. As shown in Fig. 7C-D, a significant increase in the SC pool was observed in muscles deficient for HDAC11 (old WT mice showed 26.1 ± 5.1 SCs per 100 fibers and HDAC11^−/−^ old mice showed 33.9 ± 4.0 SCs per 100 fibers), without changes in the number of proliferating SCs. Intriguingly, we did not observe changes in the number of differentiating MYOG^+^ SCs (Figure S5), but the expression analysis of muscle differentiation-related genes involved in myofiber repair (dyspherlin) and myocyte fusion (myomaker, and myomixer), showed significant higher expression in HDAC11^−/−^ regenerating muscles (Fig. 7E). Following muscle injury, infiltrating macrophages play a critical role during the skeletal muscle regeneration process, which is why we analysed the expression level of various cytokines produced by macrophages at 7dpi and our results showed higher expression of *Tnfα*, *Il-1β*, *Il-6* and *Il-10* in muscles deficient for HDAC11 (Fig. 7F).

Taken together, these results suggest that lack of HDAC11 protects ageing-associated satellite cell pool depletion. In addition, skeletal muscles deficient for HDAC11 show enhanced skeletal muscle regeneration, with a higher numbers of SCs, which may differentiate/fuse more rapidly contributing to the increased area of regenerated fibers, in an inflammation-related environment that favours skeletal muscle regeneration.

## Discussion

**Fig. 8:**
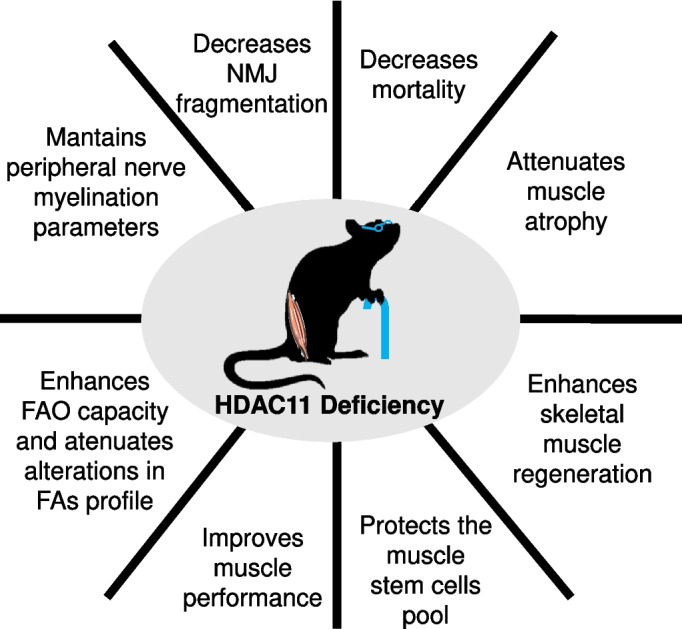
Summary of the consequences of HDAC11 deficiency in aged mice. HDAC11 deficiency affects mice by decreasing their mortality. Furthermore, muscle performance is improved in functional assays. Aged nerve myelination is not altered, however NMJ fragmentation is decreased. Aged muscle lacking HDAC11 shows enhanced FAO capacity, and age-related alterations in the FAs profile are reduced. As well, age related muscle atrophy is attenuated, and following injury muscle regeneration is improved. Notably, both in homeostasis and in injury, the muscle stem cell pool is protected

Our study evaluates for the first time the consequences of HDAC11 deficiency in skeletal muscle ageing, in homeostasis and during muscle regeneration. Skeletal muscle ageing, a process known as sarcopenia, is characterized by the progressive loss of muscle mass and reduced muscle function. Our results showed that lack of HDAC11 diminished mortality and resulted in healthier ageing by attenuating age-associated muscle atrophy, postsynaptic NMJ fragmentation, alterations in skeletal muscle fatty acid composition, preserving the muscle stem cell reservoir, enhancing muscle regeneration, and globally improving muscle function (Fig. 8).

Skeletal muscles consist of heterogeneous muscle fibers classified into slow twitch (also called type I myofibers, which display oxidative metabolism) or fast twitch myofibers (sub-classified in type IIA, type IIX and type IIB myofibers, with a predominant glycolytic metabolism) [22]. In ageing, as well as in muscle pathologies, fast-twitch myofibers are more prone to degenerate and atrophy than slow-twitch myofibers [23–25] and interestingly, it has been reported that increasing glycolytic fiber mass reverses the effects of ageing in mice [26]. Remarkably, our results showed that lack of HDAC11 reduces ageing-associated muscle atrophy, attenuating type II myofiber atrophy. The mechanisms underpinning skeletal muscle atrophy are complex, and involve an imbalance in protein synthesis, degradation pathways, and atrogene (genes whose expression is up- or down-regulated during the atrophying phase and return to normal levels during recovery [27]) expression. Our results showed lower levels of MuRF1 and CTSL atrogenes in HDAC11^−/−^ old mice compared to their WT counterparts. Notably, evidence shows that histone deacetylases are attractive targets for reducing muscle atrophy. For instance, administration of the pan HDAC inhibitor butyrate has been found to prevent muscle loss during ageing [28], and HDAC4 and HDAC5 deficient mice are protected against muscle atrophy in a surgical model of denervation [29]. In addition, it has been shown that myosin heavy chain (MyHC), PGC-1a and HSc70 proteins can be deacetylated by HDAC4 and that maintaining acetylation of MyHC and Hsc70 prevents muscle atrophy[30]. However, because the deacetylase activity of HDAC11 is considered negligible by several authors [12–14], it would be unlikely that HDAC11 deacetylates these proteins.

Sarcopenia is also linked to age-related changes in NMJs, and histones deacetylases have been reported to play a role in NMJ function. For instance, HDAC6 is a key α-tubulin deacetylase in muscle cells and controls the structure of NMJs, thereby ultimately regulating the accumulation of AChRs at postsynaptic sites in mice [31]. HDAC4 is considered an important mediator of the action of neural activity on muscle gene expression and its muscle expression is dramatically induced in response to denervation and in ALS mice [32]. Regarding HDAC11, our results didn’t show major changes in the morphological appearance of the NMJs comparing WT and HDAC11^−/−^ muscles. However, we observed a significant decrease in the fragmentation of the postsynaptic component that is still innervated in the absence of HDAC11. Fragmentation is often taken as a sign of NMJ dysfunction, which can lead to a pronounced sarcopenic phenotype [33]. In contrast, other studies reported that the progressive fragmentation of individual NMJs, such as occurs naturally with age, was not correlated with a decline of the efficacy of neuromuscular transmission at those NMJs [34]. However, based on our results which show an improved muscle phenotype in HDAC11^−/−^ mice, the reduced NMJ fragmentation observed in HDAC11-deficient muscles could be considered beneficial in maintaining the integrity of neuromuscular function and improving muscle function.

Our study addressed for the first time the impact of HDAC11 deficiency on the structural and functional integrity of peripheral nerves, and the potential contribution of HDAC11 in myelin and Schwann cell biology in aging. Our comprehensive analysis of ultrastructural data revealed no discernible differences in the number of myelinated axons, g-ratio, myelin infoldings, and axonal roundness of peripheral nerves from aged HDAC11^−/−^ mice. This indicates that the absence of HDAC11 does not influence the peripheral nervous system during ageing.

Importantly, our data showed that old HDAC11^−/−^ deficient mice have greater muscle strength and endurance, suggesting improved fitness. Our group recently showed that HDAC11 is found in mitochondria, suggesting that it may regulate mitochondrial biology [16]. Loss of mitochondrial function occurs with ageing, but our results showed increased fatty acid oxidation activity and increased expression of FAO-related genes (Acot1, Cpt1b and Ucp3) in old HDAC11^−/−^ muscles. Interestingly, it was recently shown that loss of HDAC11 in mice promotes thermogenic capacity by increasing UCP1 expression, stimulates brown adipose tissue formation, attenuates obesity and metabolic syndrome in response to high fat diet, and increases fatty acid oxidation metabolism [35, 36]. UCP3 is another member of the uncoupling proteins family, and it is highly expressed in skeletal muscle. UCP3 is more abundant in glycolytic type 2 muscle fibers (the ones protected in muscles lacking HDAC11) than in oxidative type 1 muscle fibers [37]. Notably, specific overexpression of UCP3 in skeletal muscle results in increased fatty acid uptake and oxidation capacity compared to wild-type mice [38], supporting the hypothesised role of UCP3 in facilitating fatty acid oxidation in muscle and preventing cytosolic triglyceride storage [39], which would correlate with our observation of lower lipid droplet accumulation in old HDAC11-deficient muscles. Importantly, it has recently been reported that elevated FAO is a metabolic feature in long-lived individuals, which could explain the lower mortality in HDAC11-deficient mice [40].

Our study has characterised for the first time the composition of fatty acids in aged HDAC11^−/−^ skeletal muscles. Our data showed that lack of HDAC11 attenuates age-associated changes in fatty acid composition, showing saturated, monounsaturated and polyunsaturated fatty acid percentages more similar to those found in young WT muscles. In addition, HDAC11 deficiency resulted in a significant decrease in the omega-6/omega-3 fatty acid ratio (n-6/n-3 ratio) and a dramatic increase in the omega-3 index. Omega-3 and omega-6 polyunsaturated fatty acids are essential lipids obtained through diet, and a high n-6/n-3 ratio is associated with the pathogenesis of several human diseases and with ageing [5, 6]. Interestingly, there is accumulating evidence that dietary supplementation of n-3 promotes skeletal muscle function, increases muscle mass, and enhance muscle repair [41–46]. Our results showed that lack of HDAC11 increases significantly endogenous levels of omega 3 fatty acids in muscle tissue, leading to a more favorable fatty acid profile, which would explain the improvement of muscle function.

Muscle ageing is characterized by a decline in the muscle stem cell pool (satellite cells) and muscle repair capacity [20, 21]. Our data reports for the first time the protection of age-related SCs loss in HDAC11^−/−^ mice in homeostasis and upon injury. Furthermore, our results show improvement in muscle fiber regeneration at 7 days post CTX injury in old HDAC11^−/−^ mice, as we also recently demonstrated in HDAC11^−/−^ young mice [15]. In the absence of HDAC11, we found a higher number of SCs at 7dpi (PAX7^+^ cells) which were also negative for KI67, indicating that they either exited the cell cycle to differentiate or returned to quiescence to restore the satellite cell pool. Intriguingly, we did not observe an increased number of myogenin positive cells, although we detected increased expression of repair- and fusion-related genes (dysferlin, myomixer and myomaker). All together, we propose that the higher expression of genes involved in membrane repair and cell fusion in HDAC11-deficient aged mice, together with the increased number of satellite cells in the regenerating muscles, would result in more efficient skeletal muscle regeneration.

Efficient skeletal muscle regeneration requires an optimal niche, which highly affects satellite cell mechanics as it modulates various signals required to function properly [21]. In this regard, our results showed an increase in the expression of the pro-inflammatory cytokines Tnfα, Il-1β and Il-6 in aged HDAC11 deficient mice, as well as the anti-inflammatory cytokine Il-10. It has been reported that HDAC11 is a negative regulator of the pro-inflammatory IL-1β cytokine [47] and also of the anti-inflammatory IL-10 [48–50]. This apparent opposite function of HDAC11 in regulating both kinds of cytokines could reflect a tightly regulated control of pro- and anti-inflammatory molecules to prevent uncontrolled inflammation, favouring the return to tissue homeostasis. In addition, although IL-6 has classically been considered a pro-inflammatory cytokine, subsequent research has shown that IL-6 also has anti-inflammatory effects, especially muscle-derived IL-6 [51]. Local transient production of IL-6 after stimuli has been found essential for SC proliferation and hypertrophic growth [52]. Furthermore, IL-6 is also considered a potent myokine involved in glucose oxidation, lipolysis and fatty acid oxidation [53]. Taken together, higher IL-6 levels in HDAC11^−/−^ skeletal muscles would promote muscle regeneration and reduce ectopic fat deposition by increasing lipolysis and fatty acid oxidation. Fibro-adipogenic progenitors (FAPs) are non-myogenic cells key for efficient skeletal muscle regeneration. During the initial phase of skeletal muscle regeneration, the FAP pool temporarily expands but in later phases excess FAPs are eliminated by TNFα-induced apoptosis, which could be faster in muscles lacking HDAC11 [54]. The Il-10 gene was the first HDAC11 epigenetic target identified [48–50], and it is a key factor in regulating myogenic differentiation in vivo [55], where it has been reported that its supplementation increases muscle strength, mass, and myofiber size [56]. Collectively, our results suggest that lack of HDAC11 would modify the muscle microenvironment, contributing to enhance skeletal muscle regeneration. However, further investigations would be needed to decipher the exact mechanisms causing these changes and their impact on the crosstalk between SCs, FAPs, and immune cells.

Intriguingly, HDAC11 displays a very potent defatty-acylase activity removing lipids (such as myristoyl groups) from lysines in proteins, and so far only two physiological substrates (SHMT2 and gravin-α) have been identified [14, 57]. To better understand the functions of HDAC11 in skeletal muscle it will be necessary to perform proteomic assays to identify the interactors and myristoylated substrates for HDAC11. In addition, HDAC11 is also higher expressed in testis, brain, heart, kidney and adipose tissue; therefore, by analizing a whole body knockout we can not rule out systemic changes affecting muscle tissue, which is a limitation of this study. Our results indicate that the absence of HDAC11 has a beneficial effect on aged muscle tissue. However, to determine whether our observations are direct, and to dissect HDAC11 functions in muscle tissue, future studies with skeletal muscle-specific HDAC11 knockout mice must be performed. Lastly, our study has analysed muscle samples from male mice; therefore, to propose HDAC11 as a global anti-sarcopenic target, it would be necessary to consider the gender perspective and analyze male and female mice.

In conclusion, healthy muscles are essential to maintain a good quality of life. Here we have shown for the first time the consequences of HDAC11-deficiency in sarcopenia. HDAC11 is overexpressed in aged muscle and its absence attenuates muscle fiber atrophy, NMJ postsynaptic fragmentation, muscle stem cell exhaustion and increases skeletal muscle regeneration. From a metabolic point of view, lack of HDAC11 improves fatty acid oxidation in muscle tissue and dramatically increases the omega-3 index, resulting in an overall improvement in muscle function. Taken together, our results point to HDAC11 as a new target for the treatment of sarcopenia. Importantly, selective HDAC11 inhibitors have recently been developed that could offer a new therapeutic approach to slow the ageing process.

## Materials and methods

### Animals

HDAC11 total knockout mice (HDAC11^−/−^) were generated and genotyped as described in Hurtado et al. (2021). WT and HDAC11^−/−^ C57BL/6 mice were maintained in standard cages under standard conditions: constant temperature (22 °C ± 2 °C), relative humidity (50 ± 10%), and 12-h light/dark schedule. Standard rodent food and clean water were provided ad libitum. Mice were left to age until they reach 20–22 months of age, when they were sacrificed by isoflurane inhalation followed by cervical dislocation, in accordance with the ethical approval (protocol number 10200), obtained from the Generalitat de Catalunya (Department of Territory and Sustainability Directorate General for Environmental Policies and Natural Environment, Commission of Animal Experimentation).

### Functional assessments

To test overall muscle performance and health, non-invasive muscle function tests were used: the treadmill exhaustion test performed with increasing speed (Panlab LE 8205), and the four limb-hanging test to measure maximum time that mice can sustain limb tension to oppose their gravitational force and weight. All tests were performed following the protocols described in the literature [16]. Mice were appropriately acclimatized prior to official testing. Running time, speed, and distance were recorded when the mice were unable to resume running for 3 s despite the electric shock. Work and power were calculated using the following formulas: work (SI) = body weight (kg) X gravity (9.81 m/s 2) X vertical speed (m/s X angle) X time (s), power (W) = work (SI)/time (s). For the hanging test, each mouse was given three trials, with a 2-min rest between trials, unless the mouse was able to hang for 10 min or more. If this was the case, only one trial was performed. The maximum hanging time (i.e., the longest of the trials) was used for the analysis.

### Organ harvesting and sample collection

Following cervical dislocation mice were weighed. Next, muscles and organs of interest were harvested under sterile conditions and quickly weighed. Soleus (SL), extensor digitalis longus (EDL), plantaris (PL), tibialis anterior (TA), and gastrocnemius (GC) muscles were cut in half (at the mid belly), and half was saved for molecular study, while the other half were mounted on OCT (VWR, 00411243) and flash frozen on liquid N_2_ for histological analysis. For NMJ analysis, quadriceps muscles were cut in half longitudinally and half were mounted on OCT and flash frozen in liquid N_2_, while the other half were frozen in dry-ice and save for molecular analyses. Sciatic nerves were isolated and fixated with the fixative solution (2% PFA (Electron Microscopy Sciences, 15710), 2.5% Glutaraldehyde (Electron Microscopy Sciences, 16220), 0.1 M Cacodylate buffer pH = 7.3 (Electron Microscopy Sciences, 12300)) for 15 min and then overnight at 4ºC. Next day samples were prepared for electron microscopy**,** as detailed below (in Electron Microscopy Analysis section). Organs of interest (heart, kidney, spleen and brain) were frozen for molecular analyses in dry-ice.

### Cardiotoxin injury and muscle regeneration

Injection of cardiotoxin was performed in the tibialis anterior and the gastrocnemius muscles of aged WT and HDAC11^−/−^ mice as described by the literature [15]. Muscle regeneration characterization was assessed by histology as described below and genes of interest were analysed through qRT-PCR as detailed below.

### Histologic, morphometric and immunohistochemistry analyses

Following cervical dislocation, muscles of interest were harvested, flash frozen in OCT (VWR, 00411243), and stored at −80ºC until they were cut to 10 µm thickness using a cryostat. Sections were stained with haematoxylin/eosin (H/E) to determine muscle structure.

The following antibodies were used: PAX7 (sc-81648, Sta Cruz, 1/20), MYOD (M-318, Sta Cruz; 1/50), KI67 (ab15580, Abcam; 1/100), embryonic myosin (F1.652, Developmental Studies Hybridoma Bank; non diluted), myogenin (sc12732, Sta Cruz; 1/40) and laminin (L9393, Sigma; 1/200). Images were visualized using a Leica DMI 6000 B microscope and several images were acquired for each animal. Quiescent satellite cells (SCs) were analyzed as positive for PAX7 (in uninjured muscles), proliferating SCs as positive for PAX7 + Ki67 and differentiating SCs as positive for myogenin (in CTX-injured mice) and were represented per 100 fibers. In eMHC staining, the CSA area of eMHC positive fibers was measured using the IMAGEJ software 1.54 g version (National Institutes of Health, Bethesda, MD, USA), and the same was done for measuring fiber type CSA. A minimum of 200–400 myofibers (depending on the study) was measured for animal and condition, as described in the literature [15, 16].

For analysis of fatty acid deposition, muscle sections were processed for Oil Red O (ORO) staining. Slides were fixed in 3.7% formaldehyde, rinsed in deionized water for 30 s and then stained with ORO working Solution (1.5-parts ORO Stock Solution (2.5 g of ORO dissolved in 400 mL of 99% isopropyl alcohol) and 1-part deionized water filtered through #42 Wattman paper) for 30 min at RT. The slides were then rinsed thrice and then mounted with a 10% glycerol solution and sealed using nail polish. Percent Area of stain was determined using IMAGEJ software 1.48v version (Wayne Rasband; National Institutes of Health, Bethesda, MD, USA).

For analysis of NMJ, quadriceps muscles were flash frozen in OCT compound (VWR, 00411243), and stored at − 80 °C. Serial sections of 40 µm thick were cut in a cryostat, collected on SuperFrost®Plus microscope slides (VWR, 631–0108) and fixed in 4% paraformaldehyde for 20 min at RT. Sections were permeabilized with 1% Triton X-100 in PBS and nonspecific binding was blocked with 4% BSA for 1 h. Then sections were incubated overnight with neurofilament primary antibody at 4ºC, (1/500; Merck, N5389). The next day they were rinsed and incubated for 4 h at RT with A488 secondary antibody, (1/1000; Molecular Probes, T1175) and α-bungarotoxin-TRICT, (1/1000; Life Technologies, A21202). Finally, sections were rinsed and mounted with Mowiol. The secondary antibody specificity was tested by incubation in the absence of a primary antibody. Maximal intensity projection of z-stack images was reconstructed using the ImageJ software 1.48v version (Wayne Rasband; National Institutes of Health, Bethesda, MD, USA) and several parameters were analysed to determine the degree of denervation of the NMJs. Briefly, full or partial denervation describes complete or partial postsynaptic sites lacking the opposing nerve terminal, respectively. Number of branching points of nerve terminals describes the arborization pattern complexity. The fragmentation of the NMJ was quantified by counting the number of discrete segments or “fragments” of the postsynaptic acetylcholine receptor clusters. An NMJ was considered fragmented when the postsynaptic structure was divided into 5 or more distinct fragments, as opposed to the continuous or slightly perforated appearance seen in intact NMJs. To quantify the area of the NMJs facing forward, the region occupied by nAChRs, labeled by BTX, was measured. A minimum of 30 NMJs per mice from at least three different mice of each genotype were quantified. Images were captured as z‐stacks for quantification by a Leica DMI 6000 B microscope. Data were analysed with ImageJ program (National Institute of Health, USA), as described by Vaughan and colleagues [58, 59].

### Electron microscopy analysis

Mice were sacrificed by cervical dislocation and the sciatic nerve was exposed and fixed by adding fixative solution (2% PFA (Electron Microscopy Sciences, 15,710), 2.5% Glutaraldehyde (Electron Microscopy Sciences, 16,220), 0.1 M Cacodylate buffer pH = 7.3 (Electron Microscopy Sciences, 12,300)) for 15 min and then overnight at 4ºC. The nerve is then washed in 0.1 M cacodylate and followed by osmication of the nerve by adding 1% osmium tetroxide in 0.1 M cacodylate buffer pH = 7.3 for 90 min at 4ºC. Samples were then dehydrated and they were then changed into a 50:50 mixture of Agar 100 resin: propylene oxide for 1 h at RT. The final change was into a 75:25 mixture of Agar 100 resin: propylene for 2 h at RT. Nerves were blocked in resin and left shaking overnight at RT. These nerves were re-blocked the following day with fresh resin. Transverse semithin Sects. (2 µm) from nerves were taken 5 mm from the sciatic notch and mounted on slides. Then, semithin sections were stained with toluidine blue for visualization. Nerve mosaics were imaged with a Leica Thunder Imager Tissue microscope with a 63 × objective. Ultrathin Sects. (100 nm) were cut with an ultramicrotome Leica ARTOS 3D and collected on copper grids. The sections were then stained with Reynolds' lead citrate for contrast enhancement. The stained samples were examined using a GeminiSEM 460 scanning electron microscope (SEM) equipped with a scanning transmission electron detector (STEM). Quantitative analyses of nerve area, number of axons and myelin infoldings were performed from the whole nerve using ImageJ software. The number of nerve fibers with focally infolded myelin (internal myelin loops) and the total number of fibers on each field was counted, and the percentage of nerve fibers with myelin infoldings was calculated. Myelin sheath and axon roundness, diameter and area were performed from 4 randomly selected fields of toluidine blue semithin sciatic nerve cross Sects. (100–120 nerve fibers per field, 63 × magnification) per animal with 3–7 mice analysed per genotype. Axon roundness, axon diameter, axon area and g-ratio were measured by using the ImageJ software. Axon roundness, an index which measures the circularity of axons, was calculated according to the formula [roundness = perimeter^2^/(4 × π × area)] [19]. A roundness index of 1 represents a perfect circle, and the roundness index increases as the shape of an axon in the cross section becomes less circular. All quantitative analyses were performed in a blinded manner.

### Gene expression analysis

Total RNA was extracted from tibialis anterior muscles using PureLink™ RNA Mini Kit (Ambion, Life Technologies, 12183018A), including ON-Column PureLink®DNase treatment (Invitrogen, Life Technologies, 12185010), and quantified using Nanodrop (ThermoScientific). Integrity was checked by observation of 28S and 18S ribosomic bands on 1% agarose gel electrophoresis stained with SYBR Safe DNA gel Stain (Invitrogen, Life Technologies S33102). For each sample, 500 ng of total RNA was reverse transcribed by SuperScript™IV, following the manufacturer’s protocol. Expression analysis of specific genes was quantified by fluorogenic real-time detection method using the LightCycler 480 PCR (Roche) instruments, in triplicates. Primers were designed using the Integrated DNA Technologies (IDT) tool (http://eu.idtdna.com/scitools/Applications/RealTimePCR/) and are listed in Table S1. Primer efficiency was calculated by extracting fluorescence raw data and using the Chainy tool (http://maplab.imppc.org/chainy/) and at least two different references genes were used for each experiment. We analyzed gene expression by RT-qPCR, using the LightCycler 480 PCR (Roche) instrument. Each sample was run in triplicate. Samples without reverse transcriptase during cDNA conversion were also assessed to ensure that there was no DNA contamination. Calculations were done only on samples where a minimum of two of the triplicates where within 0.5 CPs of one another, and this was made into an average. Primer efficiency was calculated and taken into account to calculate CP values with the ROCHE software (LightCycler® 480 Software). mRNA Relative Expression was calculated using Delta CT analysis (2^−∆CT^). At least two different references genes were used for each experiment.

### Mitochondrial DNA content

For total DNA extraction, frozen skeletal muscles were homogenized using a TissueRuptor (Qiagen) in the following digestion buffer: 50 mM Tris–HCl pH8; 5 mM EDTA; 1% SDS and 200 nM NaCl and 1 mg/ml Proteinase K. Then, samples were incubated at 55ºC overnight with shaking. DNA was purified by phenol/chloroform extraction and precipitated afterwards in absolute ethanol in presence of 0.4 M of sodium acetate. The pellet was washed twice in ethanol 70% and finally resuspended in TE buffer (10 mM Tris–HCl pH8 and 1 mM EDTA). DNA purity was checked and quantified by Nanodrop (ThermoScientific) and 100 ng of DNA was run on 1% agarose gel stained with SYBR Safe DNA gel Stain (Invitrogen, Life Technologies, S33102) to check DNA integrity. qPCR analyses were performed to measure the mitochondrial DNA (mtDNA) content in WT and HDAC11^−/−^ mice. mtDNA content was expressed as the ratio of the CT value from the mitochondrial-encoded *Cox2* gene to Ct values from the genomic-encoded *Sdha* gene. The primers are detailed in Table S2. Analyses were performed in the same manner as gene expression RT-qPCRs.

### Fatty acid oxidation analysis

Following protein extraction from flash frozen skeletal muscle, FAO analysis was performed using the Fatty Acid Oxidation (FAO) Assay Kit (BR00001) from Assay Genie (Dublin, Ireland) according to manufacturer’s instruction. Final values were obtained by subtracting control readings from reaction readings for each sample. The subtracted O.D. reading was proportional to FAO activity of the sample.

### Western blot analysis

Mouse tissues were homogenized using a TissueRuptor (Qiagen) in ice-cold RIPA buffer (150 mM NaCl, 1% Triton X-100, 0.5% deoxycholate, 0.1% SDS and 50 mM Tris pH7.4 supplemented with 2 µg/ml Aprotinin, 30 mM PMSF, 1 mM Sodium orthovanadate, 5 mM Sodium fluoride (NAF) and 2 µg/ml Pepstatin A). Protein lysates were obtained collecting supernatants after centrifugation at 16,000 g for 30 min at 4ºC. Protein concentrations were determined by A280 measurements using a NanoDrop™ND 1000 Spectrophotometer (Thermo Fisher Scientific, Waltham, MA, USA). Lysates were then mixed with sample buffer (31.5 mM Tris–HCl, pH 6.8; 10% glycerol; 5% β-mercaptoethanol; 1% SDS; 0.01% bromophenol blue), denatured at 95 °C for 5 min, and separated by SDS-PAGE at a constant voltage of 100 V for approximately 60 min in 1 × running buffer (25 mM Tris, 192 mM glycine, 0.1% SDS). Proteins were transferred to Immun-Blot® PVDF Membrane (Bio-Rad, Hercules, CA, USA) in Trans-Blot Turbo Transfer Buffer (Bio-Rad, Hercules, CA, USA) at 2.5 A, up to 25 V for 10 min. Membranes were stained with Ponceau S (Fluka, Sigma-Aldrich, St. Louis, MO, USA) to verify transfer quality and to quantify the total protein loaded and transferred in each sample. Membranes were placed in Intercept® (PBS) Blocking Buffer (LI-COR Biosciences, Lincoln, NE, USA) for 1 h at room temperature. Primary antibodies CTSL (Invitrogen, Ref. 172 MA1-26,774, 1:1000), MuRF1 (TIM63, Proteintech, Ref. 55,456, 1:3000) and HDAC11 (kindly provided by Dr. T.A. Mckinsey, 1:500) were diluted in blocking buffer and incubated with the blots overnight at 4 °C. Membranes were washed with TBS containing 0.1% Tween 20 (TBST) before incubation with (IRDye® 800CW Goat anti-Rabbit IgG Secondary Antibody or IRDye® 600CW Goat anti-Mouse IgG Secondary Antibody (LI-COR Biosciences, Lincoln, NE, USA)) at 1∶15,000 dilution for 1 h at room temperature. Membranes were washed with excess TBST and dried. Bands were scanned in the appropriate channels (700 nm for IRDye680 antibody, 800 nm for IRDye800 antibody) in Odyssey CLx Imaging System. To calculate the relative intensity of bands, ImageJ 1.54 g software (National Institutes of Health, USA) was used. First, images were imported in an uncompressed format and converted to grayscale. Then, the rectangular selection tool was used to delineate each band, measuring pixel density and obtaining Integrated Density (IntDen) values. To normalize intensities, the total Ponceau Red signal was used as a total protein reference. To calculate relative intensity, each IntDen value was divided by the corresponding total Ponceau Red signal, and the data were statistically analyzed by a two-tailed Student’s t test to compare differences between experimental groups. A *P value* < 0.05 was considered significant (*p < 0.05).

### Preparation and determination of fatty acid methyl esters

The tissue samples, previously thoroughly homogenized, were transferred into glass tubes for direct transesterification, weighing approximately 50–150 mg. The composition of fatty acids in the samples were determined as methyl esters after methylation reaction, using the method by Lepage and Roy (1896). Gas chromatograph (GC) analysis were performed on a Shimadzu GCMS-QP2010 Plus spectrometer (Shimadzu, Kyoto, Japan), with a Shimadzu AOC-20i autoinjector and a Shimadzu AOC-20 s autosampler. The columns used were Suprawax-280 (Teknokroma Analítica SA, Sant Cugat del Vallés, Barcelona, Spain). Data acquisition was performed by GCMSsolution software.

Fatty acid methyl esters were identified through mass spectral and by comparing the elution pattern and relative retention times of FAME with reference FAME mixture (GLC-744 Nu-Chek Prep. Inc., Elysian MN, USA). The results were expressed in relative amounts (percentage molar of total fatty acids).

### Statistics

Statistical significance was assess using the statistical package included in GraphPad software (version 9.3). Experimental groups were compared by two-tailed Student’s t test or ANOVA. Values are given as means. A *P value* < 0.05 was considered significant (*p < 0.05; **p < 0.01, ***p < 0.001; ****p < 0.0001). Detailed statistical information for each experiment is provided in the figure legends.

## Supplementary Information

Below is the link to the electronic supplementary material.

Supplementary file1 (PDF 939 KB)

## Data Availability

All data are available in the main text or the supplementary methods. The data that support the findings of this study are available from the corresponding author upon reasonable request.

## Abbreviations

AChR: Acetyl choline receptor
Acot1: Acyl-CoA thioesterase 1
Cd36: Cluster of differentiation 36
CNF: Central-nucleated fiber
Cox2: Cyclooxygenase-2
Cpt1: Carnitine O-palmitoyl transferase 1
CSA: Cross-sectional area
Ctsl: Cathepsin L1
CTX: Cardiotoxin
DAPI: 4’,6-Diamino-2-phenylindole
dpi: Days post injury
FA: Fatty acids
FAO: Fatty acid oxidation
g: Grams
FAPs: Fibro-adipogenic progenitors
Foxo1: Forkhead box protein O1
Foxo3: Forkhead box protein O3
GAPDH: Glyceraldehyde-3-Phosphate Dehydrogenase
GC: Gastrocnemius
GC-MS: Gas Chromatography-Mass Spectrometry
GusB: Glucuronidase Beta
HDACs: Histone deacetylases
HDAC11: Histone deacetylase 11
H/E: Haematoxylin and eosin
Il-1β: Interleukin 1 beta
Il-6: Interleukin 6
Il-10: Interleukin 10
J: Joules
KO: Knockout
m: Minutes
mtDNA: Mitochondrial DNA
MUFA: Monounsaturated fatty acids
MuRF1: Muscle ring finger 1
eMYHC: Embryonic myosin heavy chain
NAD^+^: Nicotinamide adenine dinucleotide
NF: Neurofilament
NMJ: Neuromuscular junction
OAZ1: Ornithine decarboxylase antizyme
PAX7: Paired-box transcription factor 7
Pgc1α: Peroxisome proliferator-activated receptor γ coactivator
PUFA: Polyunsaturated fatty acids
RT: Room temperature
RT-qPCR: Quantitative reverse transcription polymerase chain reaction
SCs: Satellite cells
SD: Standard deviation
SFA: Saturated fatty acids
Sdha: Succinate Dehydrogenase Complex Flavoprotein Subunit A
SL: Soleus
TA: Tibialis anterior
TEM: Transmission electron microscopy
Tnfα: Tumour necrosis factor alpha
Ucp3: Uncoupling protein 3
W: Watts
WB: Western Blot
WT: Wild type
