# Supplementary material for: HDAC11 deficiency regulates age-related muscle decline and sarcopenia
Source: GeroScience. 2025 Apr 12;47(4):5843–68. doi: 10.1007/s11357-025-01611-y (PMC12397452; doi:10.1007/s11357-025-01611-y)
Supplement: Supplementary file 1 — Supplementary file1 (PDF 939 KB) [file 11357_2025_1611_MOESM1_ESM.pdf]

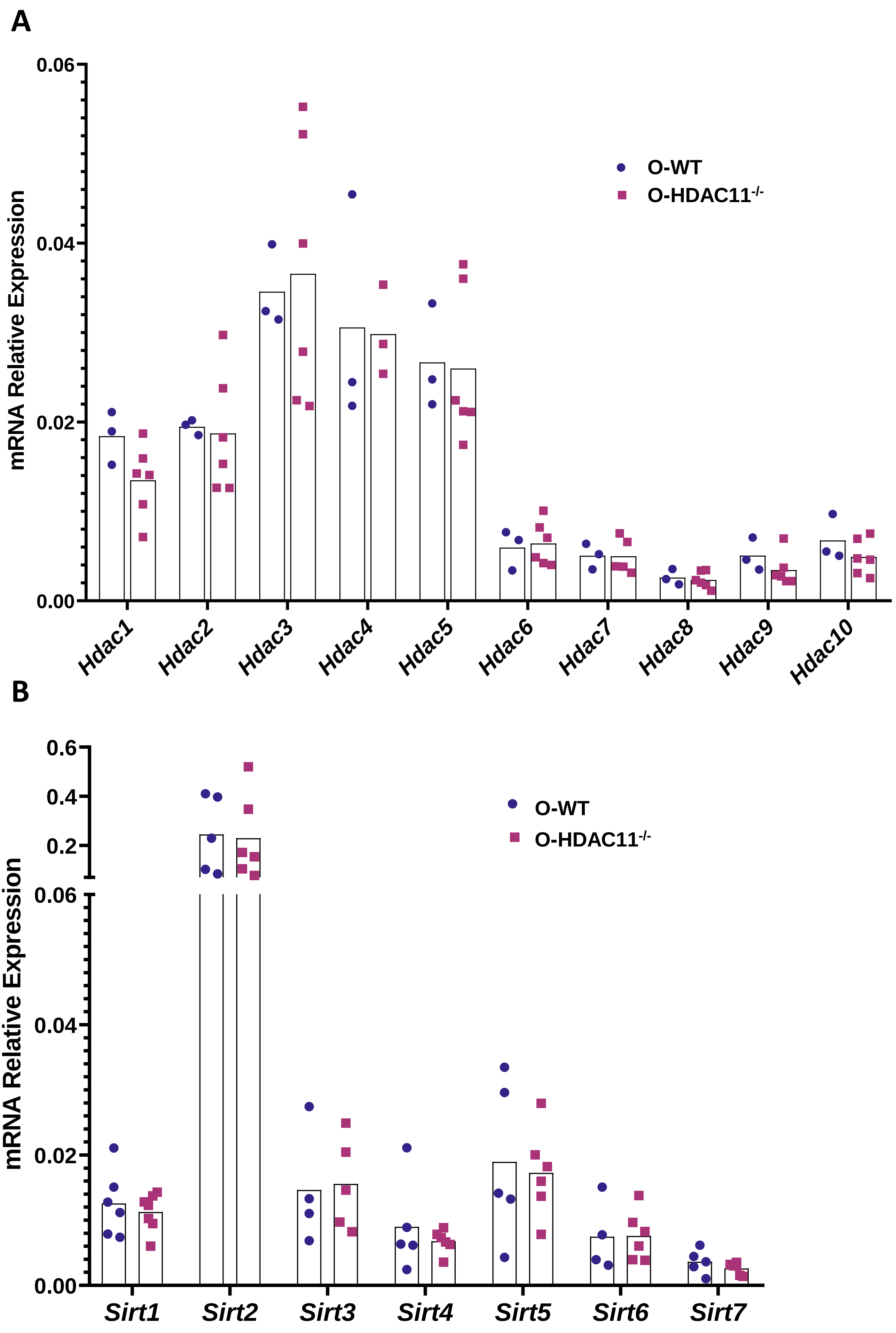

Supplemental Figure 1. Odria et al.

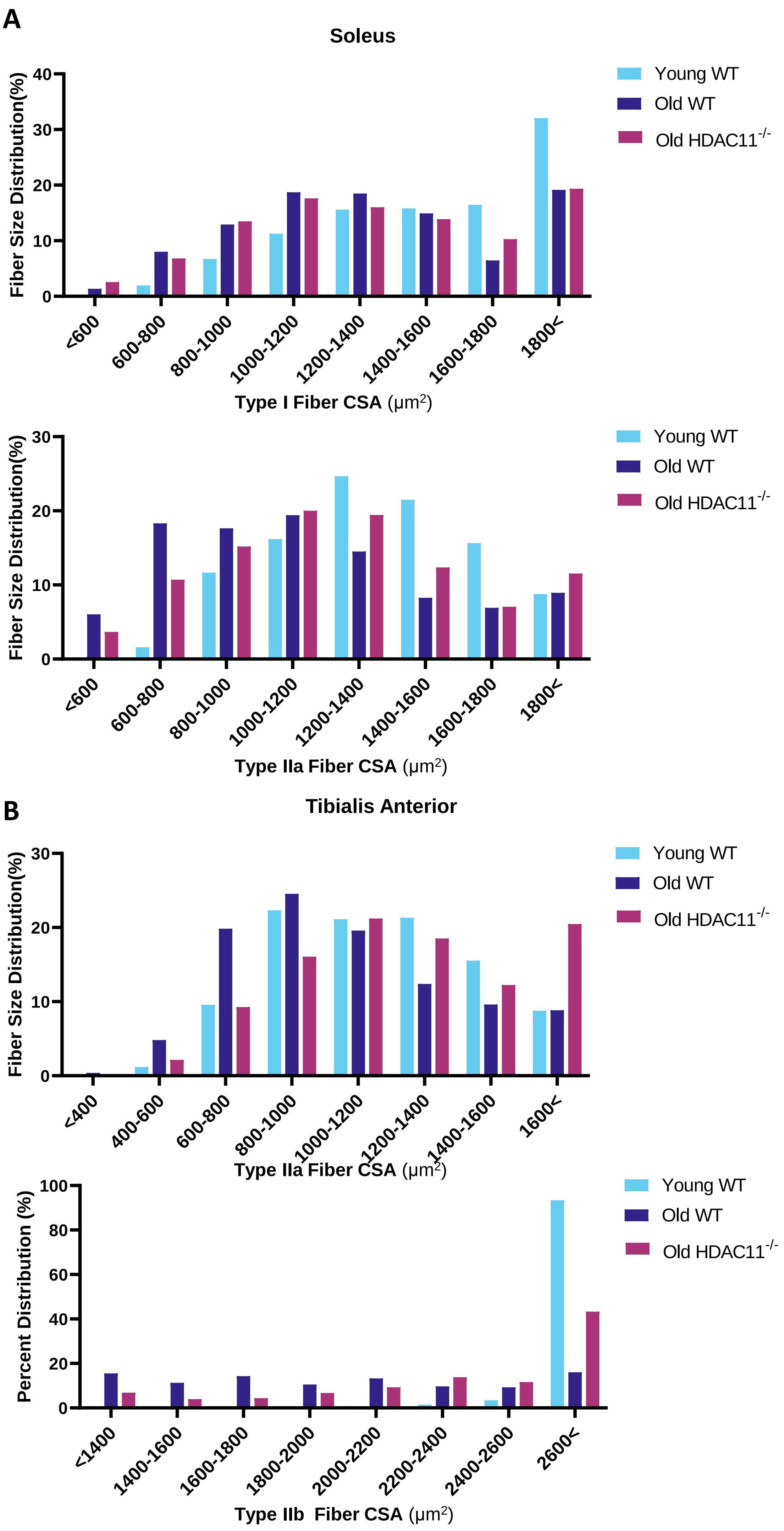

Supplemental Figure 2. Odria et al.

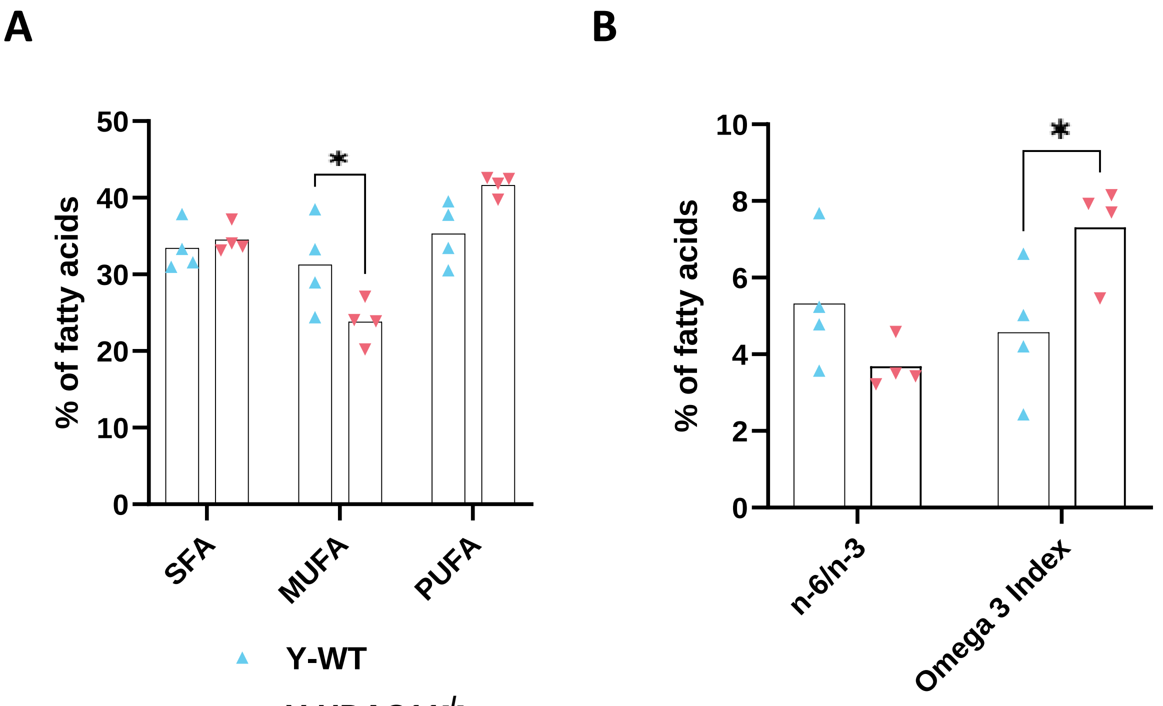

Supplemental Figure 3. Odria et al.

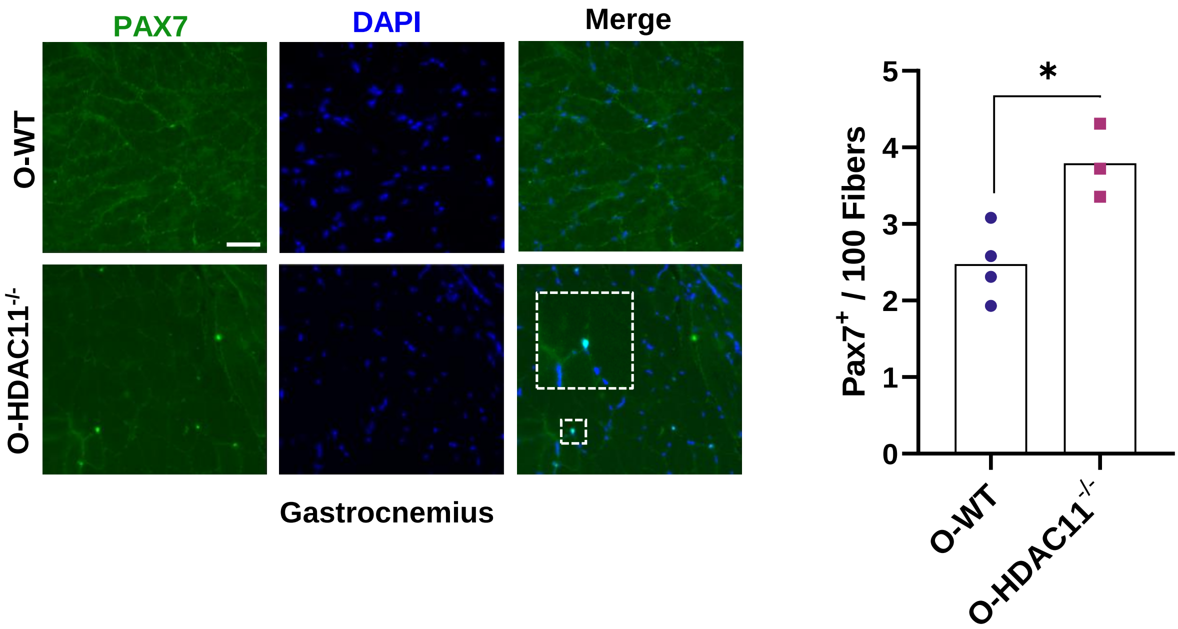

Supplemental Figure 4. Odria et al.

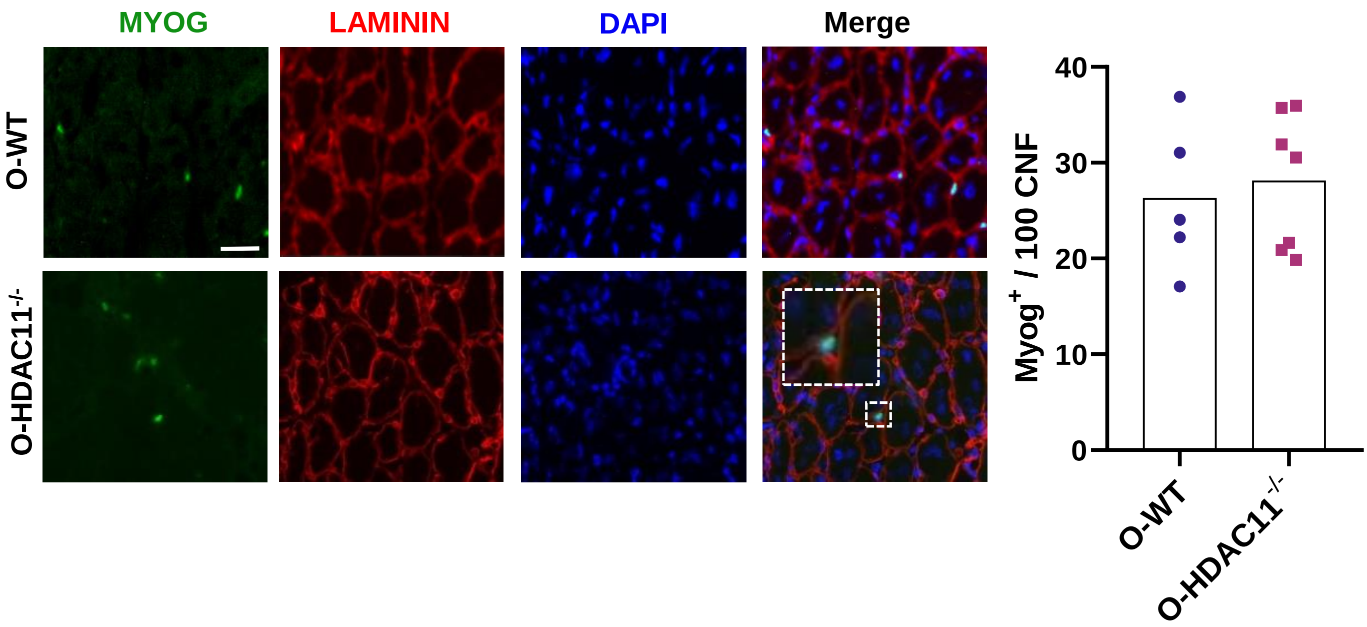

Supplemental Figure 5. Odria et al.

Supplemental Table 1. List of primer sequences used for mouse RT-qPCR analysis of mRNA expression.

Gene Name	R (5´->3´)	F (5´->3´)	Length (bp)
**Acot1**	ACTACGATGACCTCCCCAAG	CATAGCAAGGCCAAGTTCAC	149
**Bnip3**	CCTTCCATCTCTGTTACTGTCTC	ACAGCTCAGACATGAATCCTC	150
**Cd36**	GCGACATGATTAATGGCACAG	GATCCGAACACAGCGTAGATAG	115
**Cpt1b**	CCTCCGAAAAGCACCAAAAC	GCTCCAGGGTTCAGAAAGTAC	143
**Ctsl**	AGCAAGAACCTCGACCATG	TTCCATACCCCATTCACTTCC	117
**Dysf**	AGATGGACGATGCTGTGATG	CACTGAGGGTTAGCTGTCTTC	148
**FoxO1**	CTACGAGTGGATGGTGAAGAG	TGTGAAGGGACAGATTGTGG	104
**FoxO3**	CGTTGTTGGTTTGAATGTGGG	GGTTTTCTCTGTAGGTCTTCCG	143
**Hdac1**	TGAGGAGGACCCTGACAAAC	ACCACCTTCTCCCTCCTCAT	100
**Hdac2**	CATGGTGATGGTGTTGAGGA	TCATGGGAAAATTGACAGCA	151
**Hdac3**	CGACGCTGAAGAGAGAGGTC	TTTCCTTGTCGTTGTCATGG	92
**Hdac4**	TTCTGAAGCCTGCGTGTC	GGCATTGGGTCTCTGATGTAG	82
**Hdac5**	GGTTTGATGCTGTTGAAGGAC	AGATGGCGGTCAAGTCATG	150
**Hdac6**	CCTAGATGTGTCCCAACCTTG	TGTTCAGAGGCTTCATGGTG	132
**Hdac7**	CATCTGTGATGCCTCGGAG	CAGCCCCAGTATTTCCTGTG	150
**Hdac8**	ACCGAATCCAGCAAATCCTC	CAGTCACAAATTCCACAAACCG	149
**Hdac9**	CGTTCATGTAGCAATGGAAGG	GAGACTGAGGGTGTAATGGAAC	131
**Hdac10**	TTGTGTACCACGAGGACATG	CTCACAAGCTGACAAACACAG	149
**Il-1β**	ACGGACCCCAAAAGATGAAG	TTCTCCACAGCCACAATGAG	139
**Il-6**	GAACAACGATGATGCACTTGC	CTTCATGTACTCCAGGTAGCTATGGT	154
**Il-10**	CAAGGAGCATTTGAATTCCC	GGCCTTGTAGACACCTTGGTC	157
**Il-11**	TGGGACATTGGGATCTTTGC	CATTGTACATGCCGGAGGTAG	174
**MAFbx**	AGAACAGCAAAACCAAAACTCAG	GTCGAGAAGTCCAGTCTGTTG	127
**Murf1**	GCTACCTTCCTCTCAAGTGC	CCTCTGCTATGTGTTCTAAGTCC	136
**Mymk**	CATGTTCTTTGTGGCGTTCTC	CAAGCATTGTGAAGGTCGATC	179
**Mymx**	CTGTCTGCTCTTTGTCCTCAG	GTACTTTGATGGGCGTTGC	142
**Myog**	CTGCCTAAAGTGGAGATCCTG	TGGGAGTTGCATTCACTGG	182
**Pgc-1α**	CACCAAACCCACAGAAAACAG	GGGTCAGAGGAAGAGATAAAGTTG	173
**Sirt1**	CTCTGAAAGTGAGACCAGTAGC	TGTAGATGAGGCAAAGGTTCC	94
**Sirt2**	AAGGAGTGACACGCTACATG	CTTCTCCAGGTTTGCATAGAGG	134
**Sirt3**	CGGCTCTATACACAGAACATCG	CATCAGCCCATATGTCTTCCC	145
**Sirt4**	TTCCCGCTGTGGAGAGTTGC	TCAGAGTTGGAGCGGCATTGG	145
**Sirt5**	TCCCCACAAAGCAAGATCTG	TTTTCTCCAGTAACCTCCAGC	168
**Sirt6**	TTCAGCTAGAACGCATGGG	TCTTACACTTGGGACATTCCTC	141
**Sirt7**	AGGCACTTGGTTGTCTACAC	GCAGTGTCCATACTCCATTAGG	88
**Tgfβ1**	CCTGAGTGGCTGTCTTTTGA	CGTGGAGTTTGTTATCTTTGCTG	124
**Tgfβ2**	TGCTAACTTCTGTGCTGGG	TCGATCTTGGGCGTATTTCC	177
**Tnfα**	TGGAGTCATTGCTCTGTGAAG	CCTGAGCCATAATCCCCTTTC	149
**Ucp3**	CCCAACATCACAAGAAATGCC	GTCTTTACCACATCCACCGG	179
**Actb***	GATTACTGCTCTGGCTCCTAG	GACTCATCGTACTCCTGCTTG	147
**GusB***	ACTATGGGCATTTGGAGGTG	TCACTGAACATGCGAGGC	480
**Oaz1***	GCTTCATGGTCTACACTCTGG	TGGAGTGAGCGTTTATTCTGC	132
**Sdha***	AGTGCGGGTCGATGAGTATGAT	TATGAGGGGAAACGCAGGTAAG	176

***Reference Genes**

Supplemental Table 2. List of Primer sequences used for mouse qPCR analysis of mitochondrial content

Gene Name	R (5´->3´)	F (5´->3´)	Length (bp)
**Cox2**	CTACAAGACGCCACAT	GAGAGGGGAGAGCAAT	217
**Sdha**	TACTACAGCCCCAAGTCT	TGGACCCATCTTCTATGC	194

Supplemental Table 3. Description of mouse samples analysed from mice.

Technique	Sample Type	Young		Old	
		WT	HDAC11$^{-/-}$	WT	HDAC11$^{-/-}$
**WB**	QC			4	4
**Fiber analysis**	TA	4		6	9
	SL	4		6	5
**RT-qPCR**	TA			6	7
**NMJ Analysis**	QC			5	6
**Nerve**	SC			5	7
**FAO Assay**	GC			5	6
**Oil Red Staining**	SL			6	6
**mtDNA Quantification**	QC			5	5
**FA analysis**	GC	4	4	4	5
**Pax7-IHC**	TA			6	9
	GC			4	3
**eMHC - IHC**	TA			5	6
**Pax7/Ki67 and MYOG - IHC**	TA			5	7

WB: Western blot, RT-qPCR: quantitative reverse transcription PCR, NMJ: neuromuscular junction, FAO: fatty acid oxidation, mtDNA: mitochondrial DNA, FA: fatty acid, IHC: immunohistochemistry, eMHC: embryonic myosin, QC: quadriceps, TA: tibialis anterior, SL: soleus, GC: gastrocnemius, SC: sciatic nerve, WT: wild-type. All samples were from male mice.
